# Limits of Concern: suggestions for the operationalisation of a concept to determine the relevance of adverse effects in the ERA of GMOs

**DOI:** 10.1186/s12302-018-0169-6

**Published:** 2018-10-25

**Authors:** Marion Dolezel, Marianne Miklau, Andreas Heissenberger, Wolfram Reichenbecher

**Affiliations:** 1 Environment Agency Austria, Spittelauer Laende 5, 1090 Vienna, Austria; 2 Federal Agency for Nature Conservation, Konstantinstrasse 110, 53179 Bonn, Germany

**Keywords:** GMO, Genetically modified plants, Environmental risk assessment, Limits of Concern

## Abstract

**Background:**

The European Food Safety Authority proposed a concept for the environmental risk assessment of genetically modified plants in the EU that is based on the definition of thresholds for the acceptability of potential adverse effects on the environment. This concept, called Limits of Concern (LoC), needs to be further refined to be implemented in the environmental risk assessment of genetically modified organisms.

**Methods:**

We analyse and discuss how LoC can be defined for the environmental risk assessment for three different types of genetically modified plants. We outline protection goals relevant to the genetically modified plants in question and discuss existing concepts and suggestions for acceptability thresholds from the environmental risk assessment of different regulatory areas. We make specific recommendations for the setting and use of LoC for each type of genetically modified plant.

**Results:**

The LoC concept can be suitably applied for the environmental risk assessment of genetically modified organisms, if the different protection goals in agro-environments are specifically considered. Not only biodiversity protection goals but also agricultural protection goals need to be addressed. The different ecosystem services provided by weeds inside and outside agricultural fields have to be considered for genetically modified herbicide-tolerant crops. Exposure-based LoCs are suggested based on knowledge about dose–effect relationships between maize pollen and non-target Lepidoptera for insect-resistant maize. Due to the long-term nature of biological processes such as spread and establishment, LoCs for genetically modified oilseed rape should be defined for the presence of the genetically modified plant or its genetically modified traits in relevant protection goals.

**Conclusions:**

When setting LoCs, the focus should be on protection goals which are possibly affected. Potential overlaps of the LoC concept with the ecosystem service concept have to be clarified to harmonise protection levels in the agro-environment for different stressors. If additional impacts on agro-biodiversity resulting from the cultivation of genetically modified plants are to be avoided, then high protection levels and low thresholds for acceptable effects (i.e. LoC) should be set.

## Background

In the European Union, it is a crucial task and required by European legislation (Directive 2001/18/EC [1], Regulation (EC) No. 1829/2003 [2], and Implementing Regulation (EU) No. 503/2013 [3]) to assess environmental risks before introducing a genetically modified organism (GMO) into the environment or placing a product consisting of or containing GMO or parts of GMO onto the market. When carrying out an environmental risk assessment (ERA) of the GMO in question, the applicant has to follow the respective legislative provisions, in particular Annex II of Directive 2001/18/EC which lies down the principles for the ERA, and the Commission’s guidance notes supplementing Annex II [4], as well as the amendments of Annex II and III [5]. In addition, the European Food Safety Authority (EFSA) issued guidance documents for the ERA of GMOs (e.g. [6–9]). In 2010, the EFSA published a revised version of its guidance document which introduced a novel concept into the ERA of GMOs, the Limits of Concern (LoC [6]).

Limits of Concern have been introduced into the ERA requirements for GMOs to delimit those observed adverse effects which are not likely to cause environmental harm from those effects which have the potential to cause harm with respect to an identified protection goal in the agro-environment [6]. Defining environmental harm of a GMO for agro-ecosystems requires that (i) ecological entities, habitats as well as ecosystem functions and services to be preserved have explicitly been set out and (ii) their relevance for the specific GMO in question has been identified [10, 11]. Therefore, an important aspect when delimiting negligible from significant adverse effects for ERA purposes is the knowledge of what constitutes harm for defined protection goals [11]. Protection goals are “*natural resources (e.g. arthropod natural enemies, bees) or natural resource services (e.g. regulation of arthropod pest populations, pollination) that are to be protected as set out by EU legislations*” [6]. These protection goals include biodiversity, protected species and habitats, but also ecosystem functions and ecosystem services as well as different areas of protection such as water, soil and human and animal health [6]. Different approaches of how to operationalise protection goals and harm thresholds for ERA purposes have been suggested: (i) In the LoC approach suggested for the ERA of GMOs, the assessment endpoints are representatives of the relevant protection goal [6]. An assessment endpoint (e.g. a specific non-target organism or a biological function) acts as a proxy for a natural resource or resource service that requires protection and could be adversely affected by a specific stressor. Measurement endpoints are then defined for these assessment endpoints (e.g. mortality, abundance, etc.), and LoCs are set to define the level of protection [6]. (ii) In the ecosystem service approach, initially suggested for the ERA of plant protection products (PPPs) and later also for GMOs, protection goals are operationalised by identifying relevant ecosystem services and their key drivers [12, 13]. Then, specific protection goal (SPG) options are derived for each combination of key drivers and ecosystem service that may be affected [12, 13]. The formulation of SPG options also allows defining what magnitude of effect can be tolerated. The ecosystem service concept has recently been proposed for ERA use, not only for PPPs, but also for other stressors in agro-environments, such as GMOs, invasive alien species [13] or plant pests [14]. Additionally, EFSA issued two related scientific opinions that address the coverage of endangered species and the ecological recovery of non-target organisms in the ERA [15, 16]. Both documents further emphasise the need to set a common protection level for agro-ecosystems independent of the stressor.

When evaluating environmental harm due to GMO cultivation, it still has to be tackled what kind of effect is considered adverse and no longer acceptable for a particular agro-environmental protection goal. So far, generally agreed definitions and criteria about what constitutes an environmental harm exist neither for natural ecosystems nor for agro-ecosystems. For example, when assessing impacts of invasive alien species on biodiversity, either the threatening of populations of native species or the significant alteration of ecosystem processes or properties is considered as a threat to biodiversity [17, 18]. Further specifications are needed to determine, i.e. the “minimum biodiversity level for the efficient and sustainable functioning of the particular agro-ecosystem” [7]. The biodiversity of functional groups is most relevant to ecosystem functions [19]. Recently, some authors discussed what thresholds for biodiversity losses might be acceptable and whether—on a global scale—safe limits for them can be set without compromising important earth system processes [20, 21]. In addition, when setting such thresholds, this must not come into conflict with normative requirements of legislative provisions, such as for species and habitats protected at EU (e.g. FFH Directive) or national level.

A general appraisal of the LoC concept as well as suggestions for its operationalisation for ERA use has already been provided [22]. One of the basic elements of the ERA of genetically modified plants (GMP) is the comparative safety assessment [6, 9]. For the food and feed risk assessment, so-called “equivalence limits”, composed of values from commercial plant varieties with a history of safe use, are used to put any differences found into the context of harm [23, 24]. For the ERA and the establishment of environmental safety of a GMP, a similar claim does not exist when using conventional comparators (for further discussion see [22]). Using LoCs is, therefore, necessary to define whether observed differences between the GMP and the non-genetically modified (GM) comparator are biologically relevant and may cause environmental harm [6, 9, 24]. EFSA has explored the difference between biological relevance and statistical significance of an observed effect further in a scientific opinion [25]. In this opinion, expert judgment decides whether an effect is considered biologically relevant or not. This is particularly useful for the definition of LoCs for GMO risk assessment, as effects observed in the ERA are judged not only by their statistical outcome (e.g. the results of tests for difference and equivalence), but also by expert evaluation. Against this background, LoCs are understood as acceptability thresholds, either quantitatively or qualitatively, for adverse effects on biological entities, functions or processes. LoCs trigger regulatory concern due to the possibility (a) that the observed effects indicate harm to protection goals or (b) because these effects are valued as being important for specific protection goals. This definition leaves room to assign importance to observed adverse effects for which harm to the specific protection goal cannot be proven at the time being; e.g. in case that adverse effects are observed for a single species, but the consequences for the species community or the ecosystem functions provided by this species cannot be determined. Legislative provisions which define protection goals often aim to protect populations, communities or ecosystems and their services. Thus, extrapolating from effects observed at the organism level in the ERA to higher levels of biological organisation or ecosystem functions or processes is required.

**Fig. 1:**
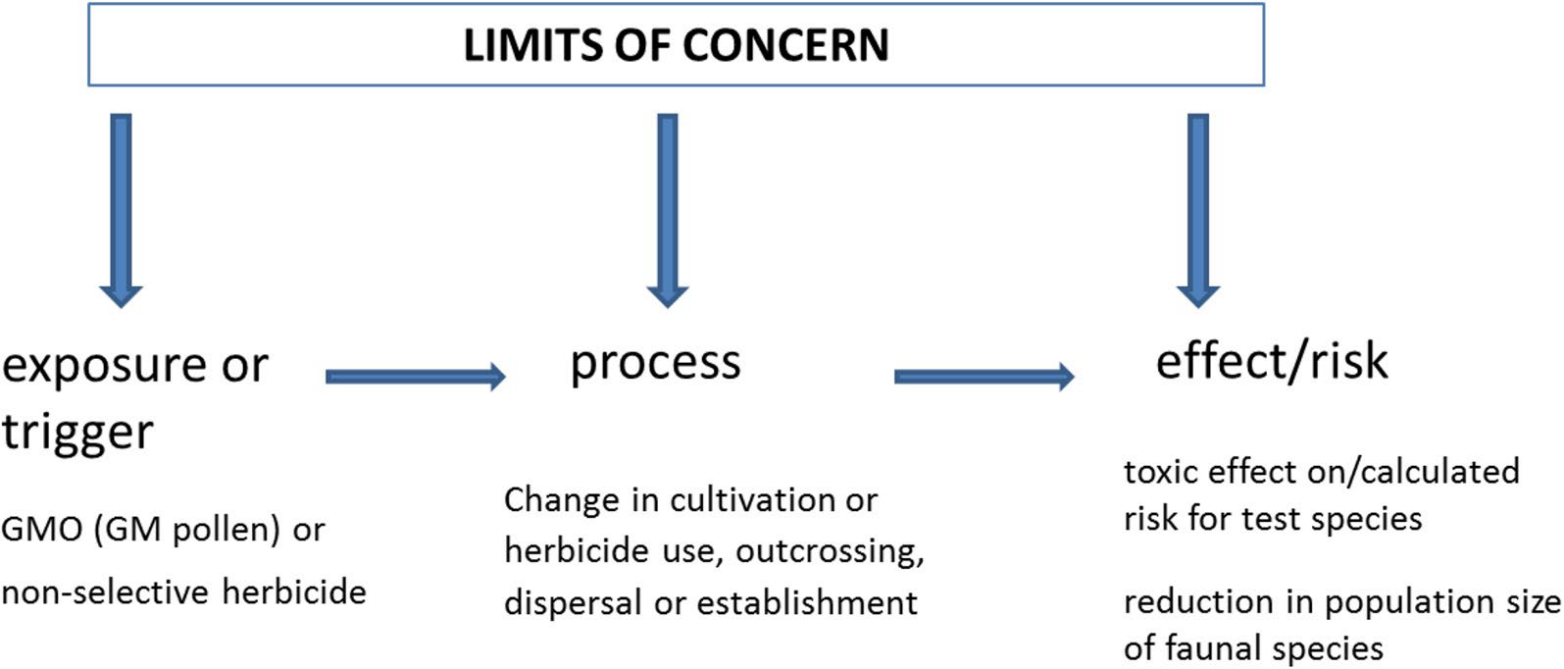
Options for setting LoCs for genetically modified plants. modified after [10]

In this article, we evaluate the LoC concept for three types of GMOs, representing three different areas of risk in the ERA according to EFSA [6]. The selection of relevant protection goals and the setting of LoCs are inherent components of the problem formulation which is the start of any ERA procedure [6]. Consequently, LoCs are principally relevant to all risk areas which have to be addressed in the ERA of a particular GMO. We base our suggestions on a conceptual framework for assessing environmental harm due to GMO cultivation elaborated by Kowarik et al. [10]. Adverse environmental effects of GMOs which impact protection goals are not always testable within the ERA, in particular in case of (i) indirect effects, e.g. effects occurring through a causal chain of events, (ii) delayed effects or (iii) effects that occur in environmental contexts not encountered during the ERA. Kowarik et al. [10] recommend the use of indicators in order to assess adverse effects of a GMO in the ERA. These indicators may be selected at different levels within a chain of adverse effects. Limits of Concern have to be set for the particular indicator chosen for the relevant protection goal. Indicators, and consequently LoCs, can be selected at the trigger or exposure level (i.e. the GMP itself or the non-selective herbicide), at the process level (i.e. processes that may lead to adverse effects) or at the effect/risk level where adverse effects are detectable (Fig. 1).

Three different types of GMOs and their specific potential environmental risks represent the respective areas of risk as outlined in the ERA guidance document by EFSA (see also “Methods”). We first determined which protection goals are relevant to each GMO. We then discuss whether thresholds for the acceptability of adverse effects with relevance for the specific GMO have already been proposed, e.g. in other regulatory areas. Finally, we address aspects that we consider most important for the setting of LoCs for each GMO. Specific links are made to the ecosystem service concept and to current developments in the ERA of GMOs or other relevant stressors in the agricultural environment.

## Results and discussion

### Example 1: GM oilseed rape

#### Relevant protection goals to be considered in the ERA of GM oilseed rape

##### Protected species

Protected species represent important protection goals for the ERA of GMPs [6]. Wild species that are able to hybridise with cultivated oilseed rape (i.e. crop-wild relatives) are part of the agro-biodiversity in agro-ecosystems. Relevant species belong to the genera *Brassica*, *Sinapis*, *Raphanus*, *Rapistrum*, *Hirschfeldia*, *Eruca* and *Erucastrum* [26]. Some of them are protected or are considered threatened, either EU-wide (e.g. [27], Annex II) or on a national scale, such as *Crambe tatarica* [28]. An important question is whether the genetic constitution of species is a relevant criterion in legislative acts. For example, the genetic constitution of species protected under the FFH Directive does not constitute a specific criterion when defining their favourable conservation status [27]. In contrast, some national provisions for nature protection (e.g. the German Nature Protection Law § 1) conserve the characteristic features of nature which may cover a specific genetic constitution [29]. The national responsibility concept includes information on the genetic constitution of populations as well [30–32].

In addition, some of the wild relatives of oilseed rape represent old crop varieties that are still cultivated in some parts of Europe, such as *Eruca sativa*, *Brassica rapa*, *Raphanus raphistrum* and *Sinapis alba* [28] and constitute important and threatened plant genetic resources [33, 34]. Wild relatives are also considered as an important breeding material for crop improvement [33]. About 18% (i.e. 25 species) of crop-wild relatives of the genus *Brassica* are considered to be threatened in Europe [33]. In-field cultivation of local, traditional and rustic breeds and varieties as well as their in situ conservation are recognised measures for the maintenance of plant genetic resources which is an important protection goal outlined in several EU-wide and international provisions for the conservation of agricultural biodiversity [35–38].

Also, crop landraces contribute to the diversity of plant genetic resources in agriculture [39], and therefore represent important protection goals for GM oilseed rape. Commission Directive 2008/62/EC defines landraces as “*a set of populations or clones of a plant species which are naturally adapted to the environmental conditions of their region*” [40]. Their conservation and sustainable use are important aims in the EU [40]. The genetic status of crop landraces and the risk of genetic erosion have been specified in the respective legislation [40]. Landraces are listed neither in the EU common catalogue of plant varieties nor in national seed catalogues, but represent an important component of Europe’s threatened agro-biodiversity [41].

##### Protected habitats

Also, habitats are biodiversity protection goals relevant to outcrossing and persistent GM crops such as GM oilseed rape, due to their ability to invade and potentially alter the species composition, structure or function of protected habitats. Habitat conservation is one of the cornerstones of the conservation of biodiversity in Europe as laid down by the FFH Directive [27] and also at national level. Feral oilseed rape occupies mainly open and disturbed habitats, either naturally or by human intervention, and establishes as a pioneer plant [42]. Some habitat types frequently occupied by feral oilseed rape, e.g. ruderal habitats that occur in agricultural landscapes, are included in national Red Lists such as the Red List of threatened habitat types in Austria [43] or the German Red Data Book on endangered habitats [44]. If GM feral oilseed rape or hybrids with wild relatives spread into these habitats, they can compromise nature conservation goals, if the specific conservation objectives of the particular habitat are adversely affected.

#### Evaluating thresholds for the acceptability of adverse effects

##### For effects on species due to vertical gene transfer of GM oilseed rape

The EFSA guidance document suggests a stepwise procedure for the ERA of GM oilseed rape, using stepwise information requirements to assess adverse effects of GM oilseed rape on wild relatives due to vertical gene transfer [6]. Stage 1 information requirements include information on hybridisation with relatives [6]. Depending on a GMP’s ability to outcross and hybridise (yes/no decision), the next stage is entered. Stage 2 should explore whether the GM trait increases the fitness of compatible relatives under agricultural conditions (yes/no decision) and—in case the answer is yes—whether environmental or agricultural impacts are evident, which can provide a link to the LoC concept. In case the GMO hybridises with compatible relatives outside production systems, further information is required in stages 3 and 4 to assess any alterations of the fitness, the range and population size of those plants. Another link to the LoC concept can be provided by evaluating whether changes in the population size of compatible relatives cause any environmental harm. However, further guidance is missing on how to evaluate environmental harm and no acceptability thresholds are proposed in the guidance document. Potential adverse effects on species and on habitats are not separately addressed.

##### For effects on habitats due to spread and establishment of GM oilseed rape

The stepwise evaluation procedure in the EFSA guidance document requires to evaluate the GMP’s ability to establish in the cropping area or outside agricultural production systems, e.g. in natural habitats [6]. In case the GMP is more persistent than its conventional counterpart, it needs to be evaluated if an agricultural or environmental impact is evident (in-field) or if environmental damage will occur outside production systems [6]. As for effects on species (see above), no further guidance is given which impacts on agricultural or natural habitats are considered acceptable.

#### Aspects to be considered when setting LoCs

Current requirements for the risk assessment of GM plants that are able to outcross and spread are based on documented harm to biodiversity. A similar way is followed when biodiversity risks of invasive alien species (IAS) are evaluated in the European Union. For both, IAS and GM oilseed rape, it is difficult to predict their spread and persistence in the environment [45] and their impact on biodiversity. Regulation (EU) No 1143/2014 on the prevention and management of the introduction and spread of IAS provides a regulatory framework with the aim to prevent or minimise threats to biodiversity in the European Community [46]. The probability of having a significant adverse impact upon biodiversity or the related ecosystem services is one of the criteria for an alien species to be listed as an IAS which also entails management actions ([46], Article 4). In this context, Essl et al. have proposed the following effects as biodiversity threats: (i) threatening of populations of native species, (ii) endangerment or extinction of native species and (iii) alteration of ecosystem processes or properties (e.g. nutrient cycling) [17]. If, according to the state of knowledge, no negative impacts to biodiversity are evident or likely, the species is considered as non-invasive and no management measures are required [17].

If similar benchmarks are also applied for GM oilseed rape, impacts on biodiversity have to be clearly evident before any thresholds for acceptability are exceeded and restrictions for cultivation or other management measures are justified. Consequently, the spread and establishment of GM oilseed rape in a natural habitat or its hybridisation with a wild relative would only constitute environmental harm if adverse impacts on biodiversity in Europe had previously been documented. For example, the hybridisation between alien species and native species is only considered as a threat to biodiversity, if effects on native populations are evident [18].

During commercial cultivation of GM oilseed rape, specific conservation objectives may be at risk in natural habitats if GM oilseed rape or GM hybrids with wild relatives establish. Whether specific conservation goals of a certain natural habitat are affected can only be decided if these are actually known. Therefore, decisions need to be taken on a case-by-case basis, considering the specific conservation objectives for the species or habitat in question. However, this procedure is not feasible within the EU-wide authorization procedure for GM crops, but must be carried out for the particular protected site in case adverse effects due to a GMP or a GM crop–wild hybrid have been observed. Similar to IAS, management action is then only required in areas of conservation concern where adverse effects have been documented and if a certain damage threshold is exceeded [17, 47].

Another approach is to define LoCs for indicators rather than for documented adverse effects on the relevant protection goals. During the ERA, several natural processes of a GM plant are assessed [6, 10]. The more of them occur (e.g. hybridisation with a wild relative, fertility of the GM crop–wild hybrid, ability to backcross into wild relatives), the higher the risk for environmental harm [10]. Similarly, for spread and establishment of a GMP or of hybrids with wild relatives, the more frequently a GMP occurs in a particular habitat, the higher is its potential for harm [10]. If spread, establishment and hybridisation are per se considered acceptable in agro-environments, they do not exceed the LoC and no risk management measures are required. This has recently been the case with the reporting of maize–teosinte hybrids in European agro-ecosystems [48–50]. Due to the GM trait conferring herbicide tolerance, the occurrence of GM maize × teosinte hybrids was considered to be restricted to agricultural fields and therefore, the potential harm considered manageable [51]. However, with other traits than herbicide tolerance, e.g. insect resistance, GM maize × teosinte hybrids may spread also to natural habitats.

**Fig. 2:**
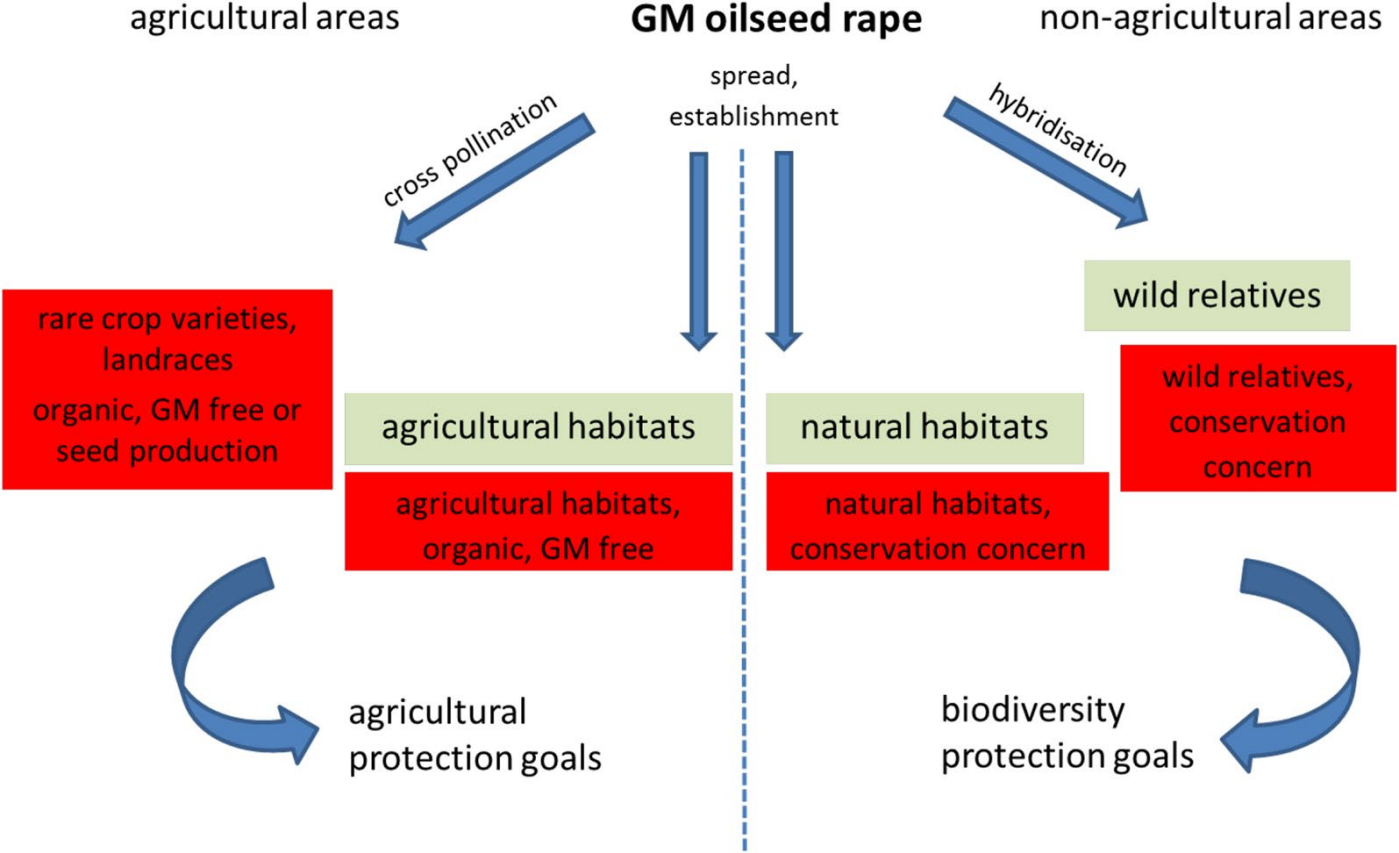
Acceptability of natural processes due to GM oilseed rape cultivation in agricultural and non-agricultural areas. The natural processes are cross-pollination, spread and establishment and hybridisation. Red boxes = agricultural and biodiversity protection goals for which the natural processes may represent unacceptable impacts. Green boxes = species and agricultural or natural habitats for which natural processes may represent acceptable impacts

These natural processes may be considered unacceptable for certain protected objects, e.g. for species or habitats of conservation concern. In this case spread, establishment or hybridisation constitutes an unacceptable process even if no impact on a native population or natural habitat is evident). Similarly, cross-pollination of GM oilseed rape with rare crop varieties, protected and endangered crop–wild species or crop landraces may be considered a threat to agricultural protection goals, and therefore valued as unacceptable (Fig. 2).

To determine whether the biological processes of hybridisation or cross-pollination are acceptable, it is required to further specify the relevant protection objectives for the protected entities in the respective laws and regulations. This includes taking decisions about the genetic condition of the respective populations of wild relatives or crop varieties to be preserved (i.e. a GM free status) or the specific genetic status to be avoided (e.g.the presence of GM constructs or traits). For landraces, the risk of genetic erosion as defined by Commission Directive 2008/62/EC [40] could be extended to specifically cover the presence of GM constructs due to cross-pollination with GM crops.

When setting LoCs, it has to be considered that GM oilseed rape—once released—is non-retrievable from the environment and that any potential adverse effect on relevant protection goals may also be non-reversible. Experience with IAS shows that eradication of already wide-spread IAS is rarely successful [52]. Other crop plants such as maize do not outcross, spread or persist without human intervention in a particular environment and can be removed once the cultivation has been terminated. Recently, experiments indicated that hybridization of cultivated maize with a weedy relative, the teosinte, can occur [50] and may, therefore, also spread and persist in the environment. It is most difficult to retrieve populations of feral crop plants such as GM oilseed rape from the environment, because (i) oilseed rape can build up long persisting soil seed banks and establish stable and self-dispersing feral populations [53, 54] and (ii) there is evidence that feral oilseed rape populations disperse beyond the arable land [55, 56]. Feral oilseed rape is expected to persist up to 20 years or even longer [57–59]. The presence of populations of GM oilseed rape in the agro-environment may, therefore, compromise relevant biodiversity and agricultural protection goals also in the long term.

### Example 2: GMHT crops

#### Relevant protection goals to be considered in the ERA of GMHT crops

##### Biodiversity protection goals

The aim of any herbicide use is to control weeds in the field where a crop is grown. This applies to GM herbicide-tolerant crops and to any other non-GM crop as well. However, rare or protected, but also common weed species require protection in agricultural landscapes [60]. It has been recognised in the ERA of PPPs that plant species growing in-field need protection as they are important for the provision of particular ecosystem services and for the support and maintenance of farmland biodiversity [61]. Weeds have been identified as key drivers for supporting food webs at higher trophic levels [61–63]. In addition, it has been recognised that certain in-field species need specific protection because they are rare or endangered [61]. Rare and endangered species play an important role as they provide aesthetic values and cultural ecosystem services in agro-ecosystems [12, 61]. Consequently, the ERA of active ingredients as well as of PPPs requires data of non-target plants not only off-field but also in-field [64, 65]. The maintenance of biodiversity is an overarching protection goal for terrestrial plants in agricultural systems [61]. In Europe, around 40 species of policy plants, i.e. plant species that are listed under European or international policy instruments, are subject to major threats by intensive arable farming [33]. At national level, Member States started different activities to promote the protection of arable plant species: (i) some Red Lists have been developed specifically for weed species (e.g. [66] for Slovakia, [67] for Poland) or for threatened biotope types including arable and fallow land (e.g. [44] for Germany; [43] for Austria); (ii) areas important for the conservation of the biodiversity of segetal plant species were identified ([68] for Austria); (iii) some Member States made suggestions which communities of arable plants to conserve ([69] for United Kingdom, [70] for Germany). An important aspect when conserving rare weeds is their in situ protection which comprises the establishment and definition of “important arable plant areas” ([69], United Kingdom), “protection fields” ([71, 72], Germany) or “biodiversity hotspots” ([68], Austria). Arable land can be an important refuge for many rare, endangered or threatened species where they grow under specific environmental or agronomic conditions [43, 68, 73]. As these species depend on the specific habitat conditions for their growth, Red Lists for endangered biotope types are also an important instrument for the conservation of arable plants [43, 44]. The previous statements underline that it is required to define protection goals also at Member State level.

##### Agricultural protection goals

For the ERA of GMHT crops, EFSA [6] refers to two legislative documents comprising agricultural protection goals: the Biodiversity Action Plan for Agriculture [35] and Directive 2009/128/EC on the sustainable use of pesticides [74]. Agricultural protection goals relevant to GMHT crops are (i) the integration of biodiversity and sustainability into agricultural production practices such as the diversification of production types and cultivated varieties (including crop rotation), (ii) less input of agrochemicals (including PPPs) and (iii) maintenance of diversity of crop varieties. Directive 2009/128/EC on the sustainable use of pesticides promotes integrated pest management with the focus on non-chemical methods and also aims at the reduction of risks and impacts of pesticide use on human health and the environment [74]. According to this Directive, EU Member States are requested to define reduction targets for pesticide use for particular crops. Another aim of the Directive is to limit the levels of pesticide use in order not to increase the risk for resistance development in populations of harmful organisms (see [74]; Annex III).

#### Evaluating thresholds for the acceptability of adverse effects

##### For effects on weeds and non-target plants

The ERA of plant protection products recommends applying the ecosystem service concept, thereby treating non-target plants differently according to their role as key drivers for different ecosystem services in-field and off-field [61].

In the field, the specific function of non-target plants, e.g. the provision of food or habitat for higher trophic levels, is considered as more important than the biodiversity of the whole plant community [61]. Consequently, the magnitude of acceptable effects for various in-field non-target plants depends on the ecosystem service they provide [61]. Medium effects are considered as acceptable for plants which act as key drivers of the food web support service, and medium to large effects for plants which are key drivers of aesthetic values and genetic resources [61].

In the off-field area, non-target plant populations are considered important drivers for a range of ecosystem services and should not be affected by the herbicides [61]. Therefore, only negligible effects are considered acceptable.

##### For effects on weed-related biodiversity

The ERA of GMOs requires an assessment whether changes in the crop management method adversely affect the biodiversity of higher trophic levels, e.g. due to changes in the weed flora [1]. Also, ERA requirements for active ingredients of PPPs demand to assess the potential impacts on biodiversity and indirect effects via alteration of the food web [64]. However, those effects have so far been left unconsidered in the ERA of PPPs [75]. Hence, no specific threshold values for indirect food web effects are available yet in the ERA of PPPs.

Any LoCs set for weed-related biodiversity effects will have to be linked to LoCsfor in-field non-target plants. The former also depend on the strength of the trophic link between plants and the biological entities representing weed-related biodiversity, e.g. different arthropod taxa, as well as the specific protection goal that needs to be considered for the individual biological entity.

For the off-field area, indirect effects on non-target arthropods by the loss of off-field host plants have been recognised as important in the ERA of PPPs [76]. Specific protection goals for non-target off-field plants have been suggested by EFSA [61] which require that they are not affected by the PPPs. If effects are not accepted on non-target off-field plants, then consequently they should not be tolerated on the arthropod community living on them.

##### For effects on agricultural protection goals

In the ERA of PPP and of GMOs, it is a clear policy goal to avoid the development of resistance in target organisms. Experience from the ERA of GMOs with insect resistance shows that even low risks for target organisms to develop resistance are not accepted and risk mitigation measures are readily proposed, such as Insect-Resistance Management plans for insect-resistant maize [77]. Although the spatial and temporal scope of the resistance development is generally not specified for insect-resistant GMOs, it can be assumed that management measures aim to avoid resistance within the EU territory, and that this requirement is, in principal, temporally not limited.

The respective legislative provisions for the ERA of PPPs and active ingredients require data on the occurrence of resistance or the possibility that target organisms develop resistance [64, 65]. Appropriate risk management strategies are required if resistance development is likely. Whether the resistance risk is deemed acceptable depends on its likelihood to occur and the possible consequences thereof [78]. In principle, the risk is considered acceptable only if a PPP can be used without any condition or restrictions and no avoidance strategy is required [78]. However, management measures can be used to sufficiently reduce the risk of resistance development, such as, e.g. specifications of a good plant protection practice, limiting the number of applications or dose rates and restrictions for the application timing [78].

When evaluating the risk that weeds develop resistance due to GMHT crop cultivation, the crop needs to be assessed together with the non-selective herbicide and compared to different weed management options currently used [6]. Risk managers decide whether risk mitigation measures are to be applied in order to avoid that weeds develop resistance to the non-selective herbicides. EFSA used risk scenarios for the assessment of glyphosate resistance occurring in GMHT soybean [79]. The relative weed-resistance risk was determined for different crop rotations and weed management options which considered the frequency of glyphosate herbicides applied to the GMHT crop, the adoption of no or reduced tillage systems and crop rotational aspects (see [79] for details). Such risk scenarios can help to determine which changes in the management regime are considered as acceptable or not and, consequently, to set the LoC.

The acceptability thresholds which have been suggested so far refer solely to the resistance risk of target organisms, but not to potential effects on any other agricultural protection goal.

#### Aspects to be considered when setting LoCs

##### Align acceptability thresholds for non-target plants used for PPPs with LoCs for GMHT crops

In the ERA of GMHT crops, thresholds for the acceptability of effects need to be considered for non-target plants in-field and off-field as applied in the ERA of PPPs. Different protection goals apply to these plants in in-field and off-field areas and therefore, different LoCs for in-field and off-field plant populations and weed-related biodiversity are necessary for GMHT crops.

##### Define different LoCs for species of conservation concern

Species of conservation concern, i.e. rare and endangered species, pose a considerable challenge in the ERA of GMPs. Since rare weed species are not commonly present in arable fields, it is challenging to assess potential adverse effects on them during ERA testing. The field scale level may not be sufficient to detect adverse effects on these species. It has been shown that the loss of less common weed species was not apparent when comparing field sites, but only with an indicator for regional upscaling [80]. In addition, rare and protected species will be underrepresented in any abundance-based selection of surrogate or focal species for ERA purposes [81].

In the USA, the decline of Monarch butterfly populations has been caused by large reductions of their larval host plant in agricultural areas with intensively grown herbicide-tolerant soybean and corn [82–84]. It cannot be ruled out that other reasons such as toxic effects through the consumption of *Bt* maize pollen and herbicide residues contributed as well. Over a time period of 11 years, host plants in agricultural fields were considerably reduced as well as the egg production of the US Midwest Monarch butterfly population [84]. It generally shows that indirect effects through agricultural practices on individual species, either alone or in combination with other adverse effects, can be severe [85].

For species of conservation concern, the magnitude of accepted effects should, therefore, be lower than for species which are not protected or endangered (see also [13]). For PPPs, it has been suggested that no adverse effects should be tolerated for in-field endangered species, also recognising that specific measures will be necessary to reach this protection goal [61]. Adverse effects on rare or threatened arable plants should be avoided to neither deteriorate their population status nor to risk their extinction [86]. The selection of these species should be based on their nomination in the following listings:

- Annex II of Council Directive 92/43/EEC on the conservation of natural habitats of wild fauna and flora [27].
- Red Lists on regional, national [66, 67, 87], European [88] or international [89] level.
- Listings for the national responsibility for the conservation of species [32].
- Listing of declining arable species [86].
- Species of special aesthetic value and representatives of cultural ecosystem services [13].
- Red Lists for endangered biotope types, e.g. [43, 44].

Rare and endangered or protected species need to be treated differently from common and widespread in-field plant species and require a different, more conservative LoC in the ERA of GMOs. Even small effects on these species should not be tolerated and will require specific risk mitigation measures. In this context, the responsibility to apply specific management measures to protect rare and endangered or protected species in agricultural fields is with the individual Member States [61].

##### Consider the role of the GMHT crop in the crop rotation

Crop rotation significantly affects weed communities and suppresses weed seed densities in comparison to monocultures (see references in [90]). Traditionally, in many European countries maize, sugar beet and oilseed rape are used as break crop for summer or winter cereals [91]. Break crops are important for sustaining certain weed populations, in particular dicotyledonous weed species, if the crop rotation is dominated by cereals ([92, 93] and references therein). If GMHT crops are used as break crops, dicotyledonous weed species are particularly adversely affected. The British Farm Scale Evaluations have shown that effects of GM maize and GM spring oilseed rape as break crops on the weed seed bank were traceable for at least two seasons after their cultivation [94]. When defining LoCs for effects on weed communities, it is important to consider a GMHT crop’s role in crop rotation, e.g. by applying a more conservative LoC for a GMHT crop that is used as break crop.

##### Choose a relevant indicator/ecological entity for the ERA before setting LoCs

It is important to choose a relevant indicator for the assessment of potential adverse effects of the GMHT crop on the weed community or higher trophic levels. Kowarik et al. [10] proposed a range of indicators for this purpose. Experience with GMHT crops from the British Farm Scale Evaluations has shown that the most prominent and significant effect of GMHT crops was on weed biomass [92, 93]. Also plant density and in particular final plant density before harvest were suitable parameters indicating substantial differences in weed management between conventional and GMHT crops. Therefore, in addition to the indicators suggested by Kowarik et al. [10], further indicators such as the reduction in weed biomass or weed density for assessing effects on in-field plant populations have been proposed [95].

There is evidence that weedy plants with specific functional traits can be affected by the non-selective herbicides and/or different types of crop management in different ways. For example, in GMHT oilseed rape, dicotyledons were significantly affected by the non-selective herbicide treatment, but monocotyledons not [93]. In addition, reproductive and non-reproductive weed species should be separately assessed, particularly at the end of the growing season, because population numbers of reproductive species indicate whether their seed bank will be sufficiently replenished at the end of the growing season. These aspects would have to be taken into account, if indicators are selected for the ERA and thresholds for the acceptability of effects are defined.

For in-field plants exposed to non-selective herbicides, it is important to consider that actually a specific weed community is the affected entity rather than the plant population of a specific taxon. However, if considering higher trophic levels and food web-related effects, the importance of a particular species may be valued higher than the weed community. This is demonstrated by the weed species *Chenopodium album* which is an important food resource for the skylark *Alauda arvensis* [96, 97]. An example from North America is the already mentioned Monarch butterfly, a species which is an obligate herbivore of milkweed species growing on agricultural land [98]. In Europe, 21 lepidopteran associations with weeds were identified and considered at high risk when cultivating GM herbicide-tolerant crops [99]. In particular, monophagous lepidopteran species occurring on weed species that cannot be controlled well by conventional herbicides were predicted to be prone to significant declines if non-selective herbicides were applied [99].

Similar to weeds, invertebrates respond to GMHT management in different ways depending on what functional group they belong to [100]. Detected adverse effects of GMHT cultivation relate to different taxonomic levels such as orders, genera and species [101–103]. For taxa at higher trophic levels (e.g. birds), adverse effects at the population level of a species may be more relevant than for the functional group. In this context, the usefulness of the ecosystem service concept is emphasised, because it allows defining the ecological entity which is to be protected, in accordance with the defined and relevant protection goal [13]. Consequently, a differentiation of the LoC may be necessary according to the ecological entity selected for the risk assessment.

##### Define weed thresholds before setting LoCs in-field

While for the ERA of PPPs, specific protection goals for off-field non-target plants have been proposed, no such protection goals have been defined for in-field plants [61]. In practice, it will be hardly feasible to differentiate—as proposed by EFSA [61]—non-target plants in the field according to their role as key driver supporting different ecosystem services. A range of plant taxa certainly supports not only a single but also several ecosystem services; therefore, individual ecosystem services may be difficult to address when protecting a particular species. In addition, there are currently no specific requirements to protect in-field non-crop plants according to current EU agricultural policies, leaving room for individual Member States to define the relevant protection level [61].

In the European Union, IPM (Integrated Pest Management) programmes and strategies have become important due to Directive 2009/128/EC on the sustainable use of pesticides ([74], Article 1). This Directive aims at the reduction of risks and impacts on human health and the environment due to pesticide use by a range of measures implemented at the national level. It encourages the use of alternative approaches to (i) reduce pesticide use, (ii) monitor pesticides and (iii) prohibit pesticides in sensitive areas. Member States are requested to promote low-pesticide-input management, in particular by implementing the principles of IPM. The IPM principles require monitoring of harmful organisms as well as the use of threshold values for decision making for plant protection measures (see [74] Annex III). Consequently, a range of countries currently develop or plan to develop crop-specific IPM guidelines [104].

In arable fields, there is a trade-off between different ecosystem services. If provisioning services such as food, feed, fibre or fuel are maximised, this often comes at the expense of others, namely regulating services (e.g. pollination or biocontrol), supporting services (e.g. soil formation) and cultural services (e.g. recreation). Balancing of the different services is, therefore, inevitable if certain protection goals are to be met [105, 106]. In this respect, weed thresholds could serve as an initial approach. They define the minimum size of weed communities required to sustain the flora and fauna that provide the regulating services.

Supporting certain levels of weed densities does not necessarily result in yield penalties or unacceptable yield reductions [105, 107]. Low-input use of pesticides and herbicides in conventional arable crops has been tested for profitability in different crops in large-scale experiments in the UK [91, 107, 108]). Weed densities at which the control costs, namely herbicide application, equal the economic return, can be calculated on the basis of weed-yield relationships [109, 110]. These specific weed densities or thresholds can then be used as a management tool. Weed–yield relationships differ between crops and locations [111] as well as cropping systems (e.g. organic and conventional [112]). Clearly, weed thresholds have to be determined for a particular crop and the specific weed community in a particular environmental and agronomic context.

##### Consider agricultural protection goals

Potential adverse effects due to the use of non-selective herbicides on agricultural protection goal(s) need to be assessed in the ERA of GMHT crops. Hence, assessment endpoints are required which reflect relevant agricultural protection goals such as the sustainable use of pesticides, reduced inputs of chemical herbicides, or the diversity of crop types within a specific crop rotation. It is important to note that the adverse effect on agricultural protection goals is mediated by a range of weed management decisions and the consequences thereof (e.g. amount and timing of herbicide applications, crop rotation, etc.). Changes in weed management decisions are difficult to assess during the pre-market ERA of GMOs as they involve a comparison with the weed management decisions in conventional crops that differ regionally and temporally throughout the EU. Current herbicide regimes in soybean or maize include the application of pre-emergence and/or post-emergence residual or foliar herbicides [77, 79, 113]. The number of post-emergence herbicide applications in conventional maize ranges from 0.4 to 2.3 depending on the country [114]. The predicted herbicide regimes for GMHT soybean or maize in the EU include two scenarios: the substituted post-emergence herbicide application (see, e.g. substitution scenario in [77, 79]) and the worst-case scenarios with different non-selective herbicide applications combined with residual herbicides [77, 79, 113].

Currently, resistance development is the sole agricultural protection goal considered in the ERA of PPPs and GMOs. Other agricultural protection goals such as crop diversity or reduction of pesticide use are relevant as well, and setting LoCs for potential effects on them should be taken into account, also considering regional differences of the receiving environment. In this context, it has to be kept in mind that some production systems (e.g. organic, IPM) have higher restrictions for applying additional and alternative herbicides and therefore, they are more vulnerable to shifts in the weed flora, e.g. due to resistant weeds.

### Example 3: *Bt* maize

#### Relevant protection goals to be considered in the ERA of *Bt* maize

##### Biodiversity protection goals

Before 2010 species of conservation concern were considered in the ERA of *Bt* maize by assessing adverse effects on the surrogate species Monarch butterfly which has no relevance for European agro-ecosystems [115]. With its ERA guidance document [6], EFSA introduced the necessity to consider potentially affected protection goals in the problem formulation of the ERA of GMOs. These protection goals refer to species and habitats listed in Annexes II and IV of the FFH Directive [27]. In 2015, EFSA proposed risk management measures for protected non-target Lepidoptera occurring in protected habitats in the agricultural landscape [116] and a year later, EFSA also outlined in detail specific aspects of how to consider endangered species when conducting the ERA [15]. Species are considered to be endangered if they are listed on Red Lists (globally, nationally or regionally) or if they are rare [15].When selecting non-target focal species for the ERA of GMOs, Hilbeck et al. [81] also recommended considering the legal protection status and the Red List status of the species as well as the national responsibility for their conservation to better address conservation aspects. For field trials, EFSA recognised that in addition to data on focal species, specific data on ecosystem services might be needed [7].

To operationalise biodiversity protection goals for the ERA of PPPs, EFSA has developed specific guidance using the ecosystem service concept as a conceptual framework [12]. Terrestrial non-target arthropods were identified as key drivers for a range of ecosystem services (e.g. pollination, pest and disease regulation, nutrient cycling, genetic resources and aesthetic values) for which specific protection goal options were defined. As EFSA favours an equal level of protection for agro-ecosystems regardless of the type of stressor and recognises the need for a harmonised approach when considering biodiversity in the ERA of regulated products [117], these considerations also apply to GMPs. In their recently developed SPG options for ERA schemes, EFSA suggested to use the ecosystem service concept also for the ERA of GMOs [13]. The suggestions specifically address non-target Lepidoptera using the case study of *Bt* maize 1507. The cultural service was identified as ecosystem service most relevant to Lepidoptera, while other services (e.g. regulating services such as pest regulation or pollination) provided by lepidopteran species were considered less relevant [13].

##### Agricultural protection goals

The Biodiversity Action Plan for Agriculture [35] emphasizes the need to integrate biodiversity and sustainability considerations into agricultural production practices. Some of the priorities are also relevant to insect-resistant GMPs, and in particular *Bt* maize, such as less agricultural inputs (including PPPs) and the avoidance of resistance development. Both priorities correspond with the European Directive 2009/128/EC on the sustainable use of pesticides which aims at limiting the levels of pesticide use in order not to increase the risk for resistance development in populations of harmful organisms ([74], Annex III). Another important aim of this Directive is to promote low-pesticide-input pest management, in particular integrated pest management (IPM).

The use of *Bt* maize may result in resistance development in the target pest or the occurrence of secondary pests. Both may lead to changes in the cultivation, management and harvesting techniques of crop plants [6]. Changes of pest control practices may indirectly affect the environment. Resistance development in target organisms has been identified as a risk in the ERA of *Bt* maize and led to the adoption of Insect Resistance Management (IRM) plans as a risk mitigation measure to delay the resistance evolution in lepidopteran target organisms when cultivating *Bt* maize [118, 119]. Avoidance of resistance development is, therefore, not only an important goal of European environmental policies, but also an implicit protection goal in the ERA of GMOs.

While the potential role of GMOs, in particular *Bt* crops, in integrated pest management is currently being investigated for European agro-ecosystems [120], it was already questioned in 2001 that *Bt* crops are at all compatible with IPM systems because they constitutively express and produce insecticidal toxins [121]. Also, experiences gained in the USA with many years of *Bt* crop cultivation show that a majority of farmers plant *Bt* maize even at predicted low pest pressure (e.g. corn rootworm or European corn borer), thereby contradicting general IPM principles [122].

Another protection goal relevant to *Bt* crops is to limit the use of applied pesticides [35, 74]. Although it is reported that under certain conditions and in certain areas, less conventional pesticides are used with *Bt* maize compared to conventional maize [123], assessments of pesticide use in the ERA of *Bt* crops should be in accordance with relevant protection goals, e.g. pesticide reduction targets, at EU and Member State level. According to Directive 2009/128/EC, Member States need to adopt National Action Plans setting national targets to reduce the risks and impacts of pesticide use on human health and the environment [74]. No aggregated information is available yet on objectives and measures set by EU Member States; however, some of the existing National Action Plans have been criticised for neither being ambitious in reducing reliance on pesticides in agriculture nor being legally binding [124, 125]. In this context, EFSA specifically refers to the consideration of “*strategic goals for the adoption of certain pest management regimes (e.g. integrated pest management and biological control)*” which could serve as the appropriate basis when comparing risks of GMPs with conventional crops. Whether insecticidal toxins produced in *Bt* crops count as pesticides [121, 126] is an additional issue in this discussion.

#### Evaluating thresholds for the acceptability of adverse effects

##### For effects assessed in laboratory studies

In the ERA of PPPs, biocides and chemical substances, but not GMOs, acceptability thresholds for toxicity/exposure ratios are commonly used as trigger values for lower tier testing (laboratory testing). If trigger values are not exceeded, they generally indicate an acceptable risk, as the predicted environmental exposure of the species is considered to be below the amount of toxin for which effects have been observed in the tests. If trigger values are exceeded, further refinements of the assessment (e.g. of the exposure values) or risk mitigation measures are required. Hence, trigger values constitute risk-based acceptability thresholds, as they indicate an acceptable risk rather than the absence of adverse effects.

There are inconsistencies in EFSA’s ERA guidelines whether the LoC should be defined (a) at the effect level or (b) at the risk level. Ad (a): According to the proposed LoC concept, the observed differences (in effects) between the GMP and its conventional counterpart are compared to the “*minimum relevant ecological effect that is deemed biologically significant* […]” [6]. This LoC needs to be aligned to the effect size that is desired to be detected by a specific statistical test design [24]. Therefore, the LoC could be considered to be set at the effect level. Ad (b): EFSA also requires that in the risk characterisation each identified risk is estimated and appropriate risk management measures shall be proposed “… *where levels of risk exceed threshold levels*” [7]. The risk management measures are needed to reduce environmental risks to levels within the LoC (see Figure 4 in [7]). This indicates that the LoC can also be based at the risk level. It is generally questionable what role LoCs should have for laboratory studies. General shortcomings of applying LoCs for laboratory toxicity testing are outlined by Dolezel et al. [22]. In addition EFSA acknowledges that “*standardised methods and models used at tier 1 levels do not measure the specific protection goals directly*” [12]. When linking the ERA with specific protection goals, EFSA recommends using a reference tier which is defined as a “*sophisticated experimental system or model that is practical for higher*-*tier use*” [12]. Consequently, it can be supposed that defined maximum tolerable effects should not be directly matched with results from testing at lower tiers such as laboratory (toxicity) testing.

##### For effects assessed in other than laboratory studies

Since 2011 non-target Lepidoptera have been addressed in the ERA of insect-resistant *Bt* maize lines (MON810, Bt11, 1507) as representatives of biodiversity and ecosystem services. In several of its scientific opinions, EFSA modelled effects on non-target Lepidoptera by *Bt* maize at local scale, i.e. on larvae within fields and their margins, and at larger scales, i.e. over a landscape and growing season [77, 116, 118, 119, 127–130]. Conclusions and risk management recommendations refer to two different protection goals: to non-target Lepidoptera occurring within fields and their margins as well as to non-target Lepidoptera of conservation concern occurring in protected habitats. EFSA has provided specific suggestions for acceptable thresholds for non-target Lepidoptera in this context.

As operational thresholds EFSA chose maximum tolerable mortalities of (a) 1% for non-target lepidopteran species not of conservation concern and (b) 0.5% for those which are of conservation concern. EFSA considered 0.5% mortality for group (b) species to be a negligible effect, while the 1% acceptable effect level was considered to be small [13]. EFSA, however, stressed that the thresholds proposed are arbitrary and “… *should be subject to amendment according to the protection goals*…” [116].

#### Aspects to be considered when setting LoCs

##### Define different LoCs for different spatial areas in agro-environments

As regards the agro-environment, a distinction needs to be drawn between areas designated for crop cultivation (in-field) and the surrounding areas (off-field, see, e.g. ERA of PPPs). The rationale for this is that the in-field and off-field areas are habitats which provide different ecosystem services. Within agricultural fields, trade-offs between production services and other services, e.g. regulating services, are evident. In case of intended off-field measures (e.g. mowing) or unintended effects from the field (e.g. spray drift), some trade-offs are also evident in off-field habitats. In the ERA of PPPs, small–medium effects are tolerable on non-target arthropods in-field, while only negligible effects on these organisms are tolerated off-field [131].

A similar distinction of the agro-environment has to be drawn when assessing adverse effects of *Bt* maize on non-target Lepidoptera. In the off-field area, non-target Lepidoptera need to be better protected than in the field where the crop is grown. Although generally few lepidopteran non-target species occur in agricultural fields [132], thresholds for the acceptability of effects still need to be determined. As with the ERA of PPPs, some effects on non-target organisms might be acceptable in the field but not off-field (see also above). In the ERA of *Bt* maize, effects on non-target Lepidoptera mostly consider species occurring in field margins and, therefore, off-field habitats. Off-field areas are considered all areas surrounding a field (e.g. hedgerows, grass strips, adjacent field, unmanaged bare land, roads) and can be considered equivalent to field margins as defined by Roy et al. [102] and referred to by EFSA for the ERA of GMOs [127]. Against this background and to harmonise protection goals for ERA purposes as required by EFSA, effects by GM crops on non-target lepidopteran species in off-field areas need to be small to negligible. Clearly, for endangered or protected Lepidoptera occurring in protected sites or biodiversity hotspots within the agro-environment [133–135], no additional adverse effects due to *Bt* maize cultivation should be tolerated at all (see also [22]).

##### Consider exposure-based LoCs for non-target Lepidoptera

Adverse effects of *Bt* maize on Lepidoptera are mediated via ingestion of the pollen by larval stages. The exposure of lepidopteran larvae, e.g. in field margins, to *Bt* maize is a suitable indicator for ERA purposes, as it directly links the potential for adverse effects with the relevant protection goal. The larval stage is the relevant entity on which the potential stressor operates [13]. Standardised methodologies for the assessment and the monitoring of lepidopteran larvae have been developed and tested for practicality [136–138]. Methods are also available for assessing and monitoring *Bt* maize pollen deposition on leaves of lepidopteran host plants [139–141].

Assessing the exposure of lepidopteran larvae to *Bt* maize pollen means that acceptability thresholds are defined at the exposure rather than at the effect or risk level. Similarly, in the regulation of ambient air pollutants, acceptability thresholds for adverse effects are defined either based on emission values or atmospheric input values [142, 143]. For example, critical loads of air pollutants are set as ecological limits for the input of atmospheric pollutants into a particular ecosystem. No adverse effects on the ecosystem are expected as long as the critical load is not exceeded (see Annex I of Gothenburg-Protocol and [142]). Exposure levels are defined as “critical levels” when they exceed levels above which adverse effects may occur on certain environmental receptors, such as trees, other plants or natural ecosystems [143]. Importantly, recommendations for critical load and critical level values for ambient air quality are based on well-established scientific knowledge such as physiological and ecological effects of nitrogen-containing pollutants on the most sensitive type of vegetation or ecosystem [144]. Hence, LoCs for *Bt* maize could also be set as exposure-based acceptability thresholds if *Bt* maize pollen is used as an exposure-based indicator.

**Fig. 3:**
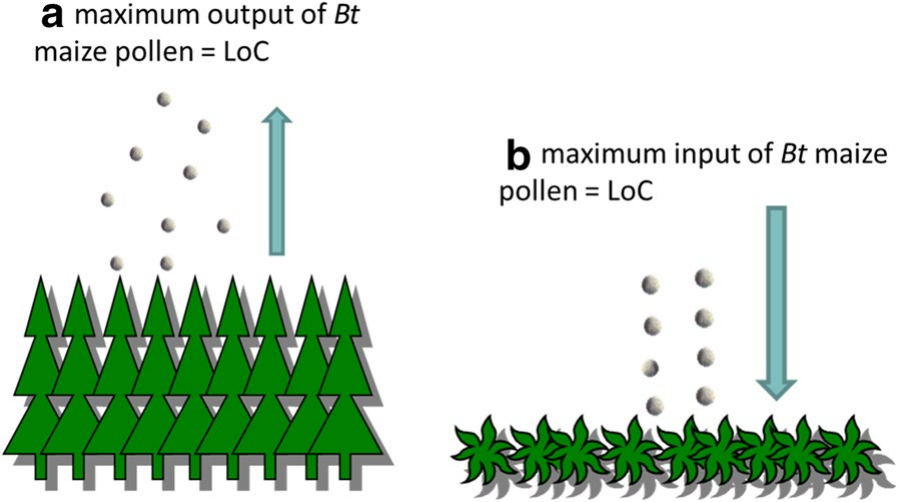
Suggestions for the operationalisation of the LoC concept for non-target lepidopteran larvae and *Bt* maize. The LoC can be defined for the exposure-based indicators (**a**) “amount of *Bt* maize pollen released” or (**b**) “amount of *Bt* maize pollen deposited” in a specific environmental reference area (e.g. the field margin). For further explanation see text

EFSA’s scientific opinion on *Bt* maize 1507 provides an example for an exposure-based threshold for *Bt* maize pollen [118]. If maize 1507 is cultivated on less than 5% of the farmed agricultural area, then the mortality for lepidopteran species was calculated to fall below an acceptability threshold of 1% and no risk management measures were required (Fig. 3a).

Another possibility is to define the maximum deposition of *Bt* maize pollen on host plants in field margins at anthesis that can be tolerated without entailing adverse effects on the larvae. It requires that dose–effect relationships of *Bt* maize pollen on non-target Lepidoptera are known and that worst-case pollen depositions are taken into account. The dose–effect relationships should be species- and toxin specific and consider that the toxin content in the pollen of *Bt* maize events can vary just as the sensitivity of non-target lepidopteran larvae. The hereof calculated maximum pollen deposition which does not harm non-target Lepidoptera constitutes a LoC at the exposure level for a specific *Bt* maize event (Fig. 3b).

Although controversially discussed, data on pollen deposition of *Bt* maize and on larval food plants under field conditions are available [141, 145–150]. Also, dose–effect relationships for lepidopteran larvae with different sensitivities to the respective *Bt* toxin from laboratory studies have been reported [118, 151–154]. It is important to notice that these relationships are based on mortalities observed under laboratory conditions. Assessment factors will, therefore, be needed to cover uncertainties, e.g. due to the extrapolation of results from the lab to the field, between species, or due to varying *Bt* toxin expression in pollen in different environments.

##### Consider the status of receiving environments when setting LoCs

The receiving environment and its specific biological and non-biological features may also have an impact on effects of GMPs. While this aspect is exemplified for *Bt* maize, it is also relevant for GM oilseed rape or other GM crops. According to EFSA, the acceptability of effects might be far less in a landscape with low non-target arthropod diversity than in a landscape supporting a high non-target arthropod diversity [131]. It is known that biodiversity levels vary considerably across European agricultural landscapes. Differences in habitat quality and habitat features in agro-environments affect species richness and abundance of butterflies [155, 156]. Species diversity of butterflies depends on the level of agricultural intensity [157] as well as on landscape context [156]. These circumstances are reflected by the geographically diverging species richness of European butterflies and by the unequal distribution of threatened and endemic butterfly species across Europe [158].

Therefore, also the natural fluctuations of lepidopteran populations need to be considered in a particular environment. Significant declines in European butterfly populations have been recorded over the last 25 years, mainly due to changes in land use including agricultural intensification [159]. The latter comes along with extensive chemical inputs such as insecticides and fungicides causing negative effects on European farmland biodiversity [160]. Considering natural fluctuations for a LoC requires defining what is the minimum environmental quality or the minimum population size to be preserved in a specific receiving environment. In this context, it is important to note that acceptability thresholds for adverse effects due to GMPs may also depend on the individual status of a population in a specific receiving environment. If a population has already declined, this may influence decisions on the acceptability of any further pressure on this population through additional stressors. In cases the reduction of a population (or its cause) is on-going, is not understood or is irreversible, a further reduction in the population size is practically unacceptable [161]. Consequently, it is necessary to define a specific environmental context, e.g. the level of biodiversity and the causes for its current status in the respective agro-ecosystem, to put the acceptability thresholds for additional adverse effects into context. However, tolerating additional impacts by GMP cultivation in environments with comparably higher biodiversity levels would still contribute to an overall reduction in biodiversity; it must be avoided and considered when setting LoCs.

## Conclusions and outlook

The issue of what constitutes environmental harm when cultivating GMP is closely linked with the question of what should be protected in European agro-ecosystems. The integration of protection goals into the environmental risk assessment carried out for stressors in the agro-environment such as GMPs is a novel feature, and the operationalisation for ERA purposes poses still unsolved challenges. The European Food Safety Authority proposed two approaches on how to operationalise protection goals in the ERA: the LoC approach and the ecosystem service approach. Both are useful but they need further specifications to be fit for purpose. In addition, overlaps between them will have to be clarified to harmonise the protection level for common protection goals in agro-environments. For example, also, the ecosystem service concept provides the opportunity to include thresholds for the acceptability of adverse effects. A major challenge is to define the relevant effect categories (e.g. negligible, small, medium, large) which are used when defining the maximum tolerable effect for a specific protection goal and align them among the two approaches. This step shall ensure that protection levels are comparable for similar protection goals for both concepts. The classification of effect categories should be transparent and relate to the specific protection goal in question.

In this context, it is important to note that the respective legislative acts for the protection and conservation of agricultural biodiversity lack some essential specifications: They do (i) neither provide benchmarks for biodiversity in arable production systems, (ii) nor define what constitutes a “good ecological status”, (iii) nor determine what environmental quality should be preserved in dynamic and resilient agro-ecosystems. In addition, knowledge of the essential ecological processes relevant to a particular ecosystem service is limited [162]. Although it has been generally postulated that any minimum level of biodiversity to sustain certain ecosystem functions can be maintained by a certain set of functionally distinct species [163], knowledge is incomplete on which and how many species and functions are actually necessary to sustain a certain ecosystem service.

When setting LoC, the regional level of farmland biodiversity in the receiving environment should be considered. This relates, if existing, e.g. to diverse field margins and natural landscape elements, the regional crop diversity and alternative food sources for higher trophic levels, since these components influence the likely magnitude of impacts on biodiversity in agricultural landscapes. Because biodiversity in agricultural landscapes as well as crop management systems highly vary across Europe, any decision on the acceptability of adverse impacts during the EU-wide ERA should consider worst-case scenarios. If adverse impacts in receiving environments with poor biodiversity are not accepted, and this forms the benchmark, then they are also not tolerated in receiving environments with a rich or more diverse biodiversity. This kind of approach would safeguard the prevention of further biodiversity losses in European agro-ecosystems.

The proposed ecosystem service concept for the ERA cannot be applied in a general manner, but requires considering different protection goals at several levels. For example, in-field and off-field habitats in the agro-environment are subject to different protection goals. Also different protection levels are assigned to biological entities, functions and processes occurring in various spatial areas of the agricultural landscape as they provide different ecosystem services. It has been acknowledged that effects outside the crop production area should not exceed negligible effects, although a specification of this effect class has still to be made for different ecological entities, functions and ecosystem services. In contrast, provisioning services in the field are often valued higher at the expense of other ecosystem services, e.g. regulating services. If the “safe ecological limits” of these other services have already been reached, needs to be assessed case-by-case for a specific stressor in a defined receiving environment. Species of conservation concern which occur in the agro-environment or within agricultural fields are difficult to assess during the ERA; however, they need higher protection levels than common and widespread species.

In contrast to biodiversity protection goals, agricultural protection goals have so far not been largely addressed in the ERA of GMPs although they are specifically outlined in the corresponding EFSA guidance documents. For insect-resistant and herbicide-tolerant GMPs, it is a relevant agricultural protection goal to avoid the development of resistant target organisms. For this protection goal, acceptability thresholds have already been implicitly set in the ERA of those kinds of GMPs, and risk management measures have already been required. In the future, defining LoCs for agricultural protection goals will necessitate additional specifications, e.g. with respect to the particular crop rotation or the relevant crop production type to be used as a baseline for the comparison with the GMO. By now, the ERA needs to account for a sustainable use of pesticides, e.g. by defining LoCs for changes in pesticide and herbicide application when cultivating GM crops. If available, national targets for pesticide use can facilitate the setting of acceptability thresholds for the use of non-selective herbicides when cultivating GM crops.

### LoCs for GM oilseed rape

In the ERA of GMOs, natural processes such as cross-pollination and hybridisation of GM oilseed rape with wild relatives or spread and establishment of GM plants in natural habitats are currently not considered adverse for the environment. However, these processes may compromise biodiversity as well as agricultural protection goals. The current risk assessment provisions are based on the evidence of impacts on biodiversity due to GM oilseed rape cultivation which is in analogy with the risk assessment of invasive alien species. However, control and eradication measures are hardly promising, once GM oilseed rape has established in the environment and threats to biodiversity are evident. Due to the ability of GM oilseed rape to independently spread and to build up soil seed reservoirs, post approval management measures, such as control of feral oilseed rape, are unlikely to succeed in the long term.

Hybridisation of GM oilseed rape with compatible wild relatives can constitute an unacceptable adverse effect if these are of conservation concern. Also, the conservation of plant genetic resources, comprising landraces, conservation varieties as well as crop–wild species, are specific agricultural protection goals, for which cross-pollination with GM oilseed rape may be unacceptable. So far, these agricultural protection goals have not been recognised in the ERA of GM oilseed rape.

Consequently, it is necessary to specify the genetic constitution of species and varieties which are able to cross-pollinate with GM oilseed rape to justify any decisions on the acceptance or non-acceptance of gene flow and hybridisation between them. Case-by-case evaluations have to clarify whether the specific conservation goals of natural habitats of conservation concern are put at risk in case GM oilseed rape spreads and establishes.

It also needs to be considered that once GM oilseed rape is approved for cultivation, hybridisation with compatible relatives, spread into and establishment in certain habitat types will occur by chance to both, protected and unprotected species and habitats. If cross-pollination and hybridisation with protected species and the occurrence or establishment of GM oilseed rape in habitats of conservation concern are considered unacceptable, then specific management measures need to be set up (e.g. the removal of the plants from the habitat). Otherwise, no such management measures will be required. In practice, however, it will be difficult to apply and manage different LoCs for species and habitats with different conservation status.

### LoCs for GMHT crops

The assessment of environmental risks of GMHT crops is covered by two distinct ERA requirements—for the GMO and for the related PPP to be used with the GMHT crop. Adverse effects of GMHT crops are mediated by the non-selective herbicide applied with the GMHT crop. Therefore, it is important to consider the GMHT crop together with its non-selective herbicide when discussing LoCs, in particular if protection goals related to biodiversity and ecosystem services are to be harmonised among different stressors in agro-environments, as intended by EFSA [13].

Weed communities play a crucial role in the assessment of adverse effects of the cultivation of GMHT crops and their weed management. In the context of the ERA of PPPs, EFSA has provided guidance for the definition of acceptable effects for weeds and non-target plants in-field and off-field. In the crop production area, there is a need for balancing different ecosystem services, such as food production and other ecosystem services. Recently, Milner and Boyd suggested a system of risk-based pesticide use to make trade-offs between environmental costs and food production more explicit; it would require, inter alia, knowledge on pesticide residues in the environment as well as setting safe environmental limits for pesticide use [164]. At smaller scales, it has been shown that such trade-offs are manageable by the ecosystem service approach [165]. Recognising that weeds are important drivers of ecosystem services also in-field, minimum levels of weed biodiversity in different crops and in different receiving environments still have to be defined to sustain the related ecosystem services. In this context, the application of weed thresholds is a useful tool to define thresholds for the acceptability of effects on weeds in agricultural fields. Consequently, LoCs for effects on higher trophic levels (e.g. arthropods or birds) in-field must be aligned to acceptability thresholds for weeds in-field, but also need to consider that the knowledge on quantitative links between weeds and higher trophic levels is largely limited [91]. In contrast to the obviously needed balancing of ecosystem services in the field, it is evident that off-field effects of herbicides used with GMHT crops need to be largely avoided.

### LoCs for *Bt* maize

For the ERA of *Bt* maize, non-target butterflies are considered important representatives of biodiversity, but they have also been attributed important ecosystem services. When assessing adverse effects on non-target Lepidoptera in laboratory studies, discrepancies are evident regarding the application of effect-based and risk-based acceptability thresholds. Using LoCs based on the exposure of non-target lepidopteran larvae to *Bt* maize pollen is a practical way to operationalise the LoC concept in the ERA of *Bt* maize. However, scrutiny will be needed whether LoCs for non-target Lepidoptera need differentiation depending on the larval stage, species, and population affected. In addition, protection levels for lepidopteran species occurring in agro-ecosystems and the relevant spatial and temporal scales need to be harmonised among the ERAs of different stressors and linked to protection goals relevant to non-target Lepidoptera.

High protection levels and low acceptability thresholds are recommended when introducing GM plants in European agricultural production systems to preserve their agro-biodiversity. When defining LoCs, it has to be considered that current agricultural practices are already compromising terrestrial and aquatic agro-biodiversity [166, 167] and, at the same time, they serve as a comparator for the evaluation of potential adverse effects due to GMO cultivation. Consequently, additional impacts on biodiversity due to GMP cultivation must not be considered acceptable in order not to further accept declines of biodiversity and ecosystem services in European agro-ecosystems.

## Methods

In 2013, the German Federal Agency for Nature Conservation commissioned the project ‘Limits of Concern for the Risk Assessment of GM plants’. The overall aim of this project was to critically evaluate the concept of Limits of Concern introduced by EFSA for its practical implementation in GMO risk assessment. A general appraisal of the concept and proposals for improvements of the general concept for use in ERA practice has been made [22]. Another aim of the project was to discuss the use of the LoC concept for specific types of GMOs.

Three different types of GMOs were used to evaluate the concept of Limits of Concern for three areas of risk which have to be assessed during the ERA of GMOs, as proposed by EFSA [6]. Using examples of GM oilseed rape, herbicide-tolerant GM crops and insect-resistant *Bt* maize, different aspects relating to the setting of LoCs were evaluated. The evaluations were based on the conceptual framework for the assessment of ecological damage for GM crops by Kowarik et al. [10]. The authors suggested a methodology for assessing ecological damage due to GMO cultivation, considering not only the potential adverse effect by the GMO, but also taking possibly affected protection goals into account [10].

In general, we evaluated EFSA guidance documents and scientific opinions for the ERA of GMOs. In addition, we consulted provisions from other regulatory areas, such as invasive alien species (for GM oilseed rape), PPPs (for herbicide-tolerant crops and *Bt* maize) as well as ambient air quality (for *Bt* maize). This was done with a view to find out whether they contain thresholds for the acceptability of environmental effects or risks which could also be relevant to GMO risk assessment.

GM oilseed rape was chosen to represent the risk area “persistence and invasiveness including plant-to-plant gene flow” according to the ERA requirements [6]. For setting LoC for this risk area, the specific GM trait is of minor relevance, as here the important aspect is the plant’s ability to hybridise with wild relatives and establish outside cultivated areas. However, considering a specific GM trait may change the potential for an adverse effect and would have to be considered separately and case-specifically. Legislative provisions regarding the ERA of invasive alien species were analysed to evaluate environmental harm thresholds for organisms that are able to spread and establish in different environmental compartments.

GM herbicide-tolerant (GMHT) plants were selected to represent the risk area “impacts of the specific cultivation, management and harvesting techniques”. In this case, the specific crop type is of minor relevance as the focus is on the potential adverse effect due to the non-selective herbicide on weeds as target organisms. For this purpose, the provisions of two different authorization regimes were considered: (i) Directive 2001/18/EC and its Annexes which require the assessment of impacts due to the specific cultivation, management and harvesting regime and (ii) Regulation (EC) No. 1107/2009 which applies for placing of plant protection products on the market [1, 168]. Aspects from the latter regulation and related documents were addressed, in case they were considered relevant to the assessment of potential adverse effects of the combined use of GMHT crops and the corresponding herbicides on weed communities and related biodiversity.

Insect-resistant (*Bt*) maize was chosen for the risk area “interactions of the GMP with non-target organisms” [6]. For this case study, the potential effects of *Bt* maize on non-target Lepidoptera were addressed. Existing EFSA guidance documents and opinions were analysed regarding suggestions for acceptability thresholds for non-target organisms, and specifically, non-target Lepidoptera. Also, legislative provisions for the regulation of ambient air quality were drawn on as an example for exposure-based acceptability thresholds.

The project benefited from three workshops conducted in 2015 for each type of GMO and each area of risk, with experts having relevant knowledge in, e.g. weed ecology, ecotoxicology of *Bt* maize, herbicide tolerance or ecology of invasive alien species. During the workshops options were discussed how to define LoCs for each particular case study.

In this article, we analyse the LoC concept for three different types of GMO intended for cultivation: GM oilseed rape, GM herbicide-tolerant crops and insect-resistant *Bt* maize. In the “Results and discussion” section, the following questions are addressed for each type of GMO in individual subchapters:

- Which protection goals are relevant to the specific GMO and area of risk?
- Have thresholds already been proposed for the acceptability of adverse effects or risks relevant to the GMO?
- Which aspects have to be taken into consideration when setting LoCs for the specific GMO in question?

## Abbreviations

EFSA: European Food Safety Authority
ERA: environmental risk assessment
GM: genetically modified
GMO: genetically modified organism
GMP: genetically modified plant
HT: herbicide tolerance
IPM: integrated pest management
LoC: Limits of Concern
PPP: plant protection product
SPG: specific protection goal
IAS: invasive alien species
